# Reliability and agreement of the timed up and go test in children and teenagers with autism spectrum disorder

**DOI:** 10.1007/s00431-023-05027-8

**Published:** 2023-05-25

**Authors:** Paloma Martín-Díaz, María Carratalá-Tejada, Francisco Molina-Rueda, Alicia Cuesta-Gómez

**Affiliations:** 1 International PhD School, Rey Juan Carlos University, 28008 Madrid, Spain; 2 Department of Physical Therapy, Occupational Therapy, Rehabilitation and Physical Medicine, Faculty of Health Sciences, Rey Juan Carlos University, Madrid, Spain

**Keywords:** Autism spectrum disorder, Balance, Gait, Reliability, Children, Measure

## Abstract

ASD patients include a variety of motor deficits; however, these issues have received less scientific attention than other ASD symptoms. Due to understanding and behavioral difficulties, it might be difficult to administer motor assessment measures to children and adolescents with ASD. To evaluate motor challenges in this population, including gait and dynamic balance issues, the timed up and go test (TUG) may be a simple, easy to apply, quick, and inexpensive tool. This test measures in seconds the time it takes for an individual to get up from a standard chair walk 3 m, turn around, walk back to the chair, and sit down again. The study purpose was to evaluate the inter- and intra-rater reliability of TUG test in children and teenagers with ASD. A total of 50 children and teenagers with ASD (43 boys and 7 girls) between 6 and 18 years were included. Reliability was verified by the intraclass correlation coefficient, standard error of measurement, and minimum detectable change. The agreement was analyzed by the Bland–Altman method. A good intra-rater reliability (ICC = 0.88; 95% CI = 0.79–0.93) and an excellent inter-rater reliability (ICC = 0,99; 95% CI = 0.98 to 0.99) were observed. Additionally, Bland–Altman plots demonstrated that there was no evidence of bias in either the replicates or between examiners. Furthermore, the limits of agreement (LOAs) between the testers and test replicates were close, indicating that there was little variation between measurements.

*       Conclusions*: The test TUG showed strong intra- and inter-rater reliability values, low proportion of measurement errors, and lack of significant bias based on by test repetition in children and teenagers with ASD. These results could be clinically useful for assessing balance and the risk of falls in children and teenagers with ASD. However, the present study is not free of limitations, such as the use of a non-probabilistic sampling.

**Table:** 

**What is Known:** • *People with ASD have a variety of motor deficits that have a prevalence rate almost as common as intellectual disability. In our knowledge, there are no studies that provide data on the reliability of the use of scales or assessment tests in children and adolescents with ASD to measure motor difficulties, such as gait and dynamic balance, in children and teenagers with ASD.* • *Timed up and go test (TUG) could be a possible tool to measure this motor skills.*
**What is New:** • *The reliability and agreement of the Timed up and go test in 50 children and teenagers with autism spectrum disorder showed strong intra- and inter-rater reliability values, low proportion of measurement errors, and lack of significant bias based on by test repetition.*

**What is Known:**

• *People with ASD have a variety of motor deficits that have a prevalence rate almost as common as intellectual disability. In our knowledge, there are no studies that provide data on the reliability of the use of scales or assessment tests in children and adolescents with ASD to measure motor difficulties, such as gait and dynamic balance, in children and teenagers with ASD.*

• *Timed up and go test (TUG) could be a possible tool to measure this motor skills.*

**What is New:**

• *The reliability and agreement of the Timed up and go test in 50 children and teenagers with autism spectrum disorder showed strong intra- and inter-rater reliability values, low proportion of measurement errors, and lack of significant bias based on by test repetition.*

## Introduction

Autism spectrum disorder (ASD) is a neurodevelopmental disorder that define a constellation of deficits in communication, social interaction, and patterns of atypical and repetitive sensorimotor behaviors [1, 2].

The etiology of ASD is multifactorial, with various genetic predispositions and environmental risk factors. [3]. Environmental risk factors include advanced parental age [4], preterm birth, low birth weight [5], short intervals between pregnancies (< 24 months) [6], or use of medications during pregnancy such as valproic acid [7, 8]. On the other hand, the genetic factors involved in ASD are still not precisely known. However, recent research has identified more than 100 genes that could be associated with the ASD phenotype [9]. In Spain, 1 per 100 school-age children have ASD. [10] Moreover, 1/100 children are diagnosed with ADS around the world [11].

ASD can be diagnosed by behavioral observation and the use of The American Psychiatric Association’s Diagnostic and Statistical Manual of Mental Disorders (DSM-5) criteria. According to DSM-5, ASD could be classified by level of severity: grade 1 (needs help), grade 2 (needs significant help), and grade 3 (needs very significant help). Therefore, DSM-5 makes a difference between the severity of social communication difficulties and restricted and repetitive behaviors that should be evaluated independently. In this classification, it is also important to specify if it is associated with other disorders like intellectual deficit; language impairment; known medical condition, genetics, or environmental factor; other neurodevelopmental, or behavioral disorder or catatonia [2]. Moreover, Revised Autism Diagnostic Interview (ADI-R), Autism Diagnostic Observation Scale-2 (ADOS-2), or childhood autism rating scale (CARS2) are examples of gold standard diagnostic scales that should be used to guarantee the accuracy of the ASD diagnosis. These scales can be used to assess the severity of ASD as well [1].

Despite the most outstanding characteristics of ASD are related to deficiencies in communication and social interaction, the evidence indicates that people with ASD also have a variety of motor deficits that have a prevalence rate almost as common as intellectual disability [12]. Some of these motor deficits are as follows: delay in fine and gross motor skills, postural instability due to possible difficulties using sensory information, altered muscle tone, difficulties in coordination, and gait disturbance [13]. To perform proficiently basic motor skills and engage in a variety of physical activities, good balance is essential. Children who have difficulty with their balance have a higher risk of falling, get fewer opportunities to learn advanced sports skills, and have difficulty engaging in physical activities [14].

Although some of these motor deficits are recognized in DSM-5 as “diagnostic-supporting associated features” such as awkward walking, clumsiness, and other anormal motor signs (for example: toe walking), these motor difficulties have not received as much research attention as communication impairments, social interaction, or intellectual disability [2, 12].

Furthermore, there are several studies that point out the strong relationship between motor skills and social skills. Likewise, there is strong evidence that the delay in achieving motor milestones is one of the characteristics that children with ASD have in the earliest stages of development [12, 13, 15, 16]. In addition, the impairments in postural control affect the development of motor and social skills in individuals with autism spectrum disorder [17].

Although it is true that earlier studies have used assessment scales to evaluate motor problems in ASD [12, 15, 18] or as an outcome measure of treatment programs for motor problems [19, 20], to the best of our knowledge, there are no studies that provide data on the reliability of the use of scales or assessment tests in children and adolescents with ASD to measure motor difficulties, such as gait and dynamic balance, in children and teenagers with ASD.

Timed up and go test (TUG) could be a possible tool to measure this motor skills. This tool has been used in clinical practice in children and teenagers to assess gait and dynamic balance [21]. This tool was originally developed with the purpose of evaluating basic motor skills in frail elderly patients [22]. In addition, it has also proven to be a good tool for evaluating functional mobility in pediatric population, presenting good reproducibility and correlation with other evaluation test [21, 23–25]. Additionally, it is a quick, simple test that does not require special equipment or training for its administration [22].

Exploring the psychometric properties of TUG test in this type of population would improve the scientific rigor of its clinical use in children with ASD.

This study aimed to examine the psychometric properties of the TUG test for measuring balance and fall risk in children and adolescents with ASD and to determine the inter- and intra-rater agreement of the TUG test in this population and obtain the measurement error (minimum detectable change) of the test.

## Materials and methods

### Participants

This study was conducted using a non-probabilistic convenience sampling. Voluntary participation of subjects with ASD was requested, subjects were recruited from different educational centers of the Madrid Community. All participants’ parents and participants older than 14 years old expressed their informed consent. The data were also coded to ensure the participants' privacy and confidentiality. Subjects were selected according to the following criteria: diagnosis of ASD, age between 6 and 18 years old, ability to follow verbal commands (score ≥ 60 on the verbal comprehension and working memory subscales of the Wechsler Intelligence Scale-IV), ability to walk at least 10 m independently and ability to sit and stand independently with or without walking aids or orthoses. Participants were excluded if they had diagnosis of any cardiovascular, musculoskeletal, respiratory, or metabolic disease, or other conditions that may interfere with this study according to their legal guardians’ reports.

### Instrumentation

**Fig. 1:**
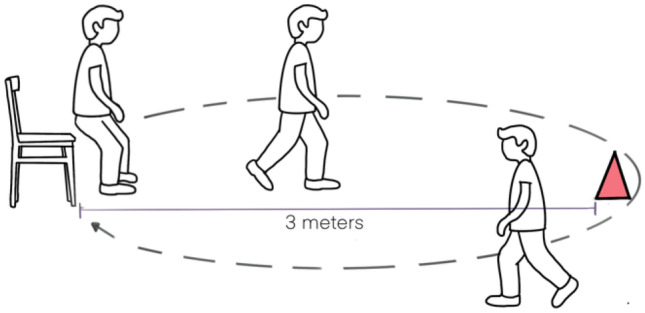
Timed up and go test

The modify version of Timed up and go test (TUG), proposed by Williams et al. [25] for children and teenagers, was used. This test was developed by Podsiadlo et al. in 1991 to assess basic motor skills in frail elderly [22]. This test measures in seconds the time it takes for an individual to get up from a standard chair walk three meters, turn around, walk back to the chair, and sit down again (Fig. 1). Better functional mobility is obtained with less time spent. The modifications to the standard TUG test were the following: instructions were repeated during the test, a seat with a backrest but without arms was selected from the children´s environment, the seat height was acceptable if the child´s knee angle was 90º, with a standard deviation (SD) of 10º, flexion with feet flat on the floor, children were allowed to behave spontaneously, so no qualitative instruction were given to ensure a naturalistic performance and timing was started as the child left the seat, rather than on the instruction “go,” and stopped as the child´s bottom touched the seat, in order to measure movement time only [25].

### Procedure

**Fig. 2:**
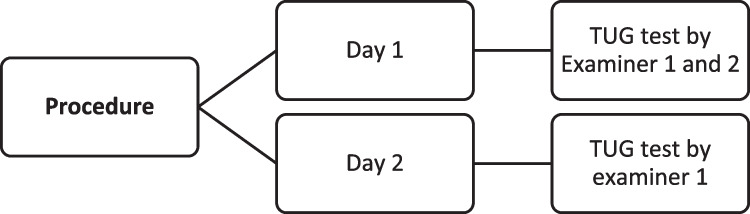
Procedure

To carry out this study, participants were evaluated by the following protocol. All the evaluations were carried out between January and September 2022:

- For inter-rater reliability, a first evaluation was carried out by two examiners who evaluated TUG test simultaneously (DAY 1). This test had an average duration of 2 min (Fig. 2).
- For intra-rater reliability, two measurements were made by the same evaluator on different days. (DAY 1 and DAY 2) According to Germanotta et al. [26–28] the distance between the evaluations were more than 1 day and less than 3 days. This test had an average duration of 2 min (Fig. 2).

All the tests were carried out with comfortable clothes that allow movement, and parents or legal representatives were allowed to be present.

All sanitary measures were taken to guarantee hygiene and safety in the COVID-19 context: mandatory mask use, frequent hand washing, material disinfection and personal distancing for as long as possible.

### Statistical analysis

Sample size was calculated based on Walter et al. [29]. The sample size was determined using the interclass correlation coefficient (ICC) and the number of raters. Considering a minimally acceptable ICC (p0) of 0,6, and an expected ICC (p1) of 0,8, and following the contingency tables of Walter et al., the necessary sample size was 43 subjects are needed. Considering a percentage of losses of 10%, the total size of the sample was established in 50 subjects.

Statistical analysis was carried out using SPSS software for Windows (SPSS Inc., Chicago, IL, USA; version 26.0). The Shapiro–Wilk test was used to screen all data for normality of distribution. Participant data and their respective times in TUG test were described by the mean and SD.

To assess the reliability between two measurements and between examiners, the ICC was used [30]. The ICC was estimated, and its 95% confidence intervals were calculated, based on absolute agreement and mixed effects model. ICC values were interpreted as excellent (> 0.90), good (0.76 − 0.90), moderate (0.50 − 0.75), and low (< 0.50).

In addition, we calculated the estimate of the standard error of the measurement (SEM), the minimum detectable change (MDC), and the inter-rater differences (SDdiff). The SEM and MDC were calculated using the following equations: $SEM=SDdiff\sqrt{(1-ICC}$) and $MDC=1.96*\sqrt{2 }*SEM$ [31].

A Bland–Altman analysis [32, 33] with 95% limits of agreement (LOA) was performed to assess intra- and inter-rater reliability. The presence of significant bias was tested by a one-sample *t* test applied to the difference between measurements. If a significant difference is observed with this test (*p* < 0.05), there is no agreement between measurements. Once the agreement between the measurements was confirmed, the Bland–Altman plots were made showing the means and the 95% LOA.

## Results

The study group consisted of 50 children and teenagers with ASD (43 boys and 7 girls; age 9.50 ± 3.086) (Table 1).

**Table 1:** Sample description

Variable	Sample size (*n* = 50)	
Age (years)	9.50 (± 3.086)	
Sex	86% (*n* = 43)	14% (*n* = 7)

The TUG test measurements showed a mean of 8.69 s with a SD of ± 1.47 s, 8.73 s (SD ± 1.48), and 8.68 s (SD ± 1.58) for examiner 1, examiner 2, and re-test respectively. The age range of 6 to 9 years showed a mean of 8.91 s (SD ± 1.4), 9.01 s (SD ± 1.42), and 8.95 s (SD ± 1.54) for examiner 1, examiner 2, and re-test respectively. In the range of 10 to 13 years showed a mean of 8.21 s (SD ± 1.39), 8.19 s (SD ± 1.33), and 8.04 s (SD ± 1.45) for examiner 1, examiner 2, and re-test respectively. In addition, the range of 14 to 18 presented a mean of 8.62 s (SD ± 1.84), 8.58 s (SD ± 1.82), and 8.75 s (SD ± 1.85) for examiner 1, examiner 2, and re-test respectively (Table 2).

**Table 2:** Description of variables stratified by age ranges

Variables		Total (*n*=50)	6−9 years (*n*=29)	10−13 years (*n*=13)	14−18 years (*n*=8)
Sex	Boys	86% (*n*= 43)	50% (*n*=25)	20% (*n*=10)	16% (*n*=8)
	Girls	14% (*n*=7)	8% (*n*=4)	6% (*n*=3)	0% (*n*=0)
TUG time (seconds)	Examiner 1	8.69 (±1.47)	8.91 (±1.41)	8.21 (±1.39)	8.62 (±1.84)
	Examiner 1	8.73 (±1.48)	9.01 (±1.42)	8.19 (±1.33)	8.58 (±1.82)
	Re-test (examiner 1)	8.68 (±1.58)	8.95 (±1.54)	8.04 (±1.45)	8.75 (±1.85)

*TUG* Timed Up and Go test, data expressed as mean ± standard deviation

* *p*-value < 0.05

The intra-rater reliability for the TUG test showed a good correlation presented an ICC = 0,88 (95% CI = 0.79 to 0.93). The range of 6 to 9 years presented an ICC = 0.84 (95% CI = 0.92 to 0.66), the range of 10 to 13 years presented an ICC = 0.84 (95% CI = 0.95 to 0.50) and the range of 14 to18 years presented an ICC = 0.98 (95% CI = 0.99 to 0.92) (Table 3).

**Table 3:** Intra-rater reliability of the timed up and go test

TUG time (seconds)			Intra-rater reliability		
Age (years)	Examiner 1	Re-test	ICC	CI 95%	*ρ*
Total	8.69 (±1.47)	8.68 (±1.58)	0.881	0.79 to 0.93	<0.001*
6-9	8.91 (±1.41)	8.95 (±1.54)	0.841	0.92 to 0.66	<0.001*
10-13	8.21 (±1.29)	8.04 (±1.45)	0.847	0.95 to 0.50	<0.001*
14-18	8.62 (±1.84)	8.75 (±1.85)	0.983	0.99 to 0.92	<0.001*

*TUG* Timed Up and Go Test, *ICC* Intraclass Correlation Coefficient, *CI* Confidence Interval. Data expressed as mean ± standard deviation

* *p*-value < 0.05

The inter-rater reliability was excellent, presented an ICC = 0,992 (95% CI = 0.986 to 0.996). The range of 6 to 9 years presented an ICC = 0.99 (95% CI = 0.996 to 0.981), the range of 10 to 13 years presented an ICC = 0.98 (95% CI = 0.997 to 0.963), and the range of 14 to 18 showed and ICC = 0.99 (95% CI = 1 to 0.994). What is more, an SEM of 0.02 was identified between examiners, which represents 0% of the mean timed observed, with an MDC of 0.06 s. The range of 6 to 9 years presented an SEM of 0.02 s with an MDC of 0.07, an SEM of 0.03 s and an MDC of 0.09 were displayed for the age range of 10 to 13 years and the range of 14 to 18 showed an MDC of 0.01, with a SEM of 0.004 s (Table 4).

**Table 4:** Inter-rater reliability of the timed up and go test

TUG time (seconds)			Inter-rater reliability			SEM	MDC
Age (years)	Examiner 1	Examiner 2	ICC	IC 95%	*ρ*		
Total	8.69 (± 1.47)	8.73 (± 1.48)	0.992	0.986 to 0.996	< 0.001*	0.02	0.06
6 − 9	8.91 (± 1.41)	9.01 (± 1.42)	0.991	0.996 to 0.981	< 0.001*	0.02	0.07
10 − 13	8.21 (± 1.29)	8.19 (± 1.33)	0.989	0.997 to 0.963	< 0.001*	0.03	0.09
14 − 18	8.62 (± 1.84)	8.58 (± 1.82)	0.999	0.997 to 0.963	< 0.001*	0.004	0.01

*TUG* Timed Up and Go test, *ICC* Intraclass Correlation Coefficient, *CI* Confidence Interval, *SEM* Standard Error of the Measurement, *MDC* Minimal Detectable Change. Data expressed as mean ± standard deviation

*p-value < 0.05

**Fig. 3:**
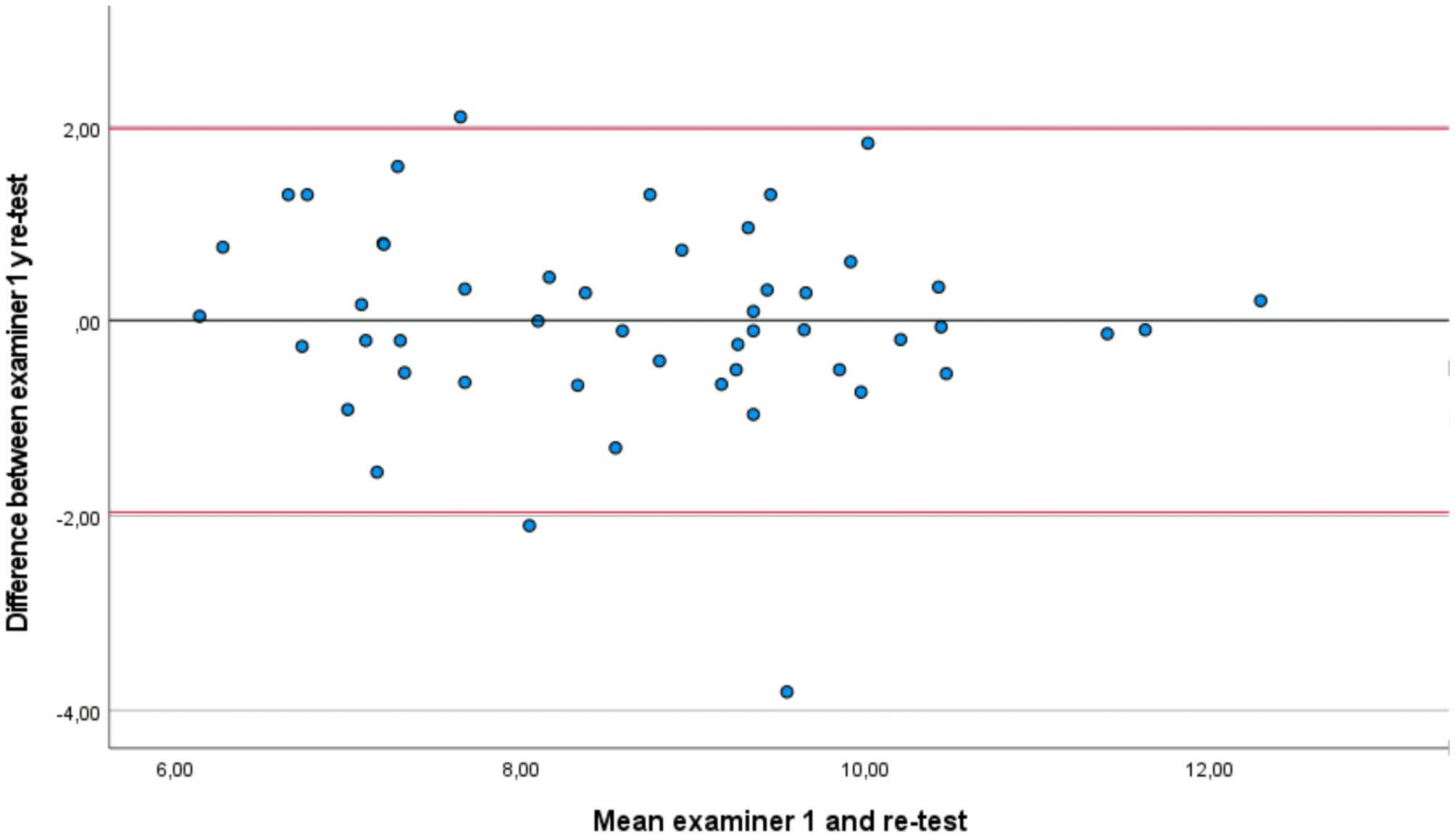
Bland–Altman plots comparing results between measurements for examiner 1 (intra-rater reliability). Bias (black line) and limits of agreement (LOAs) (red lines). The mean score is plotted on the x-axis, and the difference between measurements (mean of the differences) is plotted on the y-axis (mean difference ± 1.96 standard deviation)

**Fig. 4:**
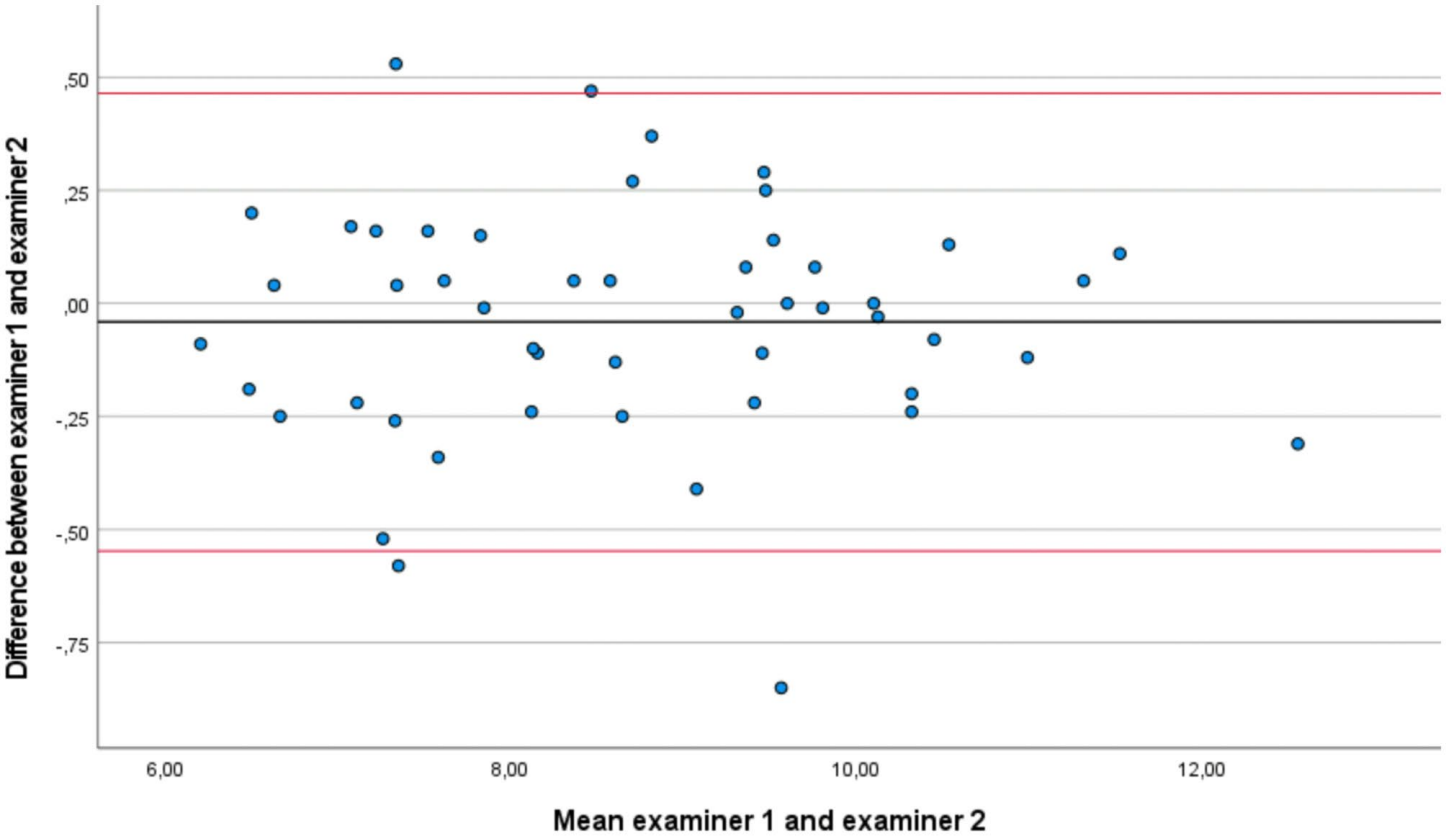
Bland–Altman plots comparing results between examiner 1 and examiner 2 measurements (inter-rater reliability). Bias (black line) and limits of agreement (LOA) (red lines). The mean score is plotted on the x-axis, and the difference between measurements (mean of the differences) is plotted on the y-axis (mean difference ± 1.96 standard deviation)

According to the Bland–Altman method (Figs. 3 and 4), there is no evidence of bias between repetitions for the TUG test (*t* = 0.07, *p* = 0.47) and between examiners (*t* =  − 1,12, *p* = 0.13). The LOA between repetitions ranging from 1.98 to − 1.96. Moreover, the LOA between examiners ranges from 0.47 to − 0.55.

## Discussion

The purpose of the current study was to evaluate the intra- and inter-rater reliability of the TUG test in children and teenagers with ASD. Results show that TUG test has good intra-rater reliability an excellent inter-rater reliability. These results could be clinically useful for assessing balance and the risk of falls in children and teenagers with ASD.

Even though it is not the only tool capable of detecting motor impairments in children with ASD, the TUG test is very easy to apply, quick, inexpensive and does not require a high-level comprehension [22], which is affected in many children and teenagers with ASD [2]. Green et al. used the Movement Assessment Battery for Children to analyze the motor impairments that children with autism have and their relationship to IQ; however, not all children were able to finish it due to the challenge of administering this type of scales to children with ASD. Due to the ease of administration in the current study, all the children evaluated completed the test [18]. The results obtained in the present reliability study are very useful to justify its clinical use in children with ASD to assess balance and gait in a functional activity.

Moreover, when compared to other test, such as those performed in a gait laboratory or hospital clinic, the TUG test could be considered as a reasonably ecologically valid instrument because it consists of a typical integrated or linked movements that are commonly performed in the child’s surroundings [25].

Previous studies have analyzed the psychometric properties of TUG test in healthy children and teenagers [21, 24, 25, 34, 35], children with cerebral palsy (CP) [35–37], spina bifida [25], traumatic brain injury [38], and Down’s Syndrome [21, 39]. However, to our knowledge, this is the first study that examine the inter- and intra-rater reliability of TUG test in children with ASD.

Williams et al. [25] previously conducted research to determine whether the TUG was a suitable test for children without disabilities and for young people with physical disability due to CP or spina bifida. The results show that TUG had a good reliability within-sessions and moderate test–retest reliability (ICC = 0.61) in children without disability aged 3 to 9 years. On the other hand, TUG showed a good within-session reliability in young people with disabilities [25]. Furthermore, Carey et al. [36] conducted a study involving a total of 51 participants with CP and found high inter-rater reliability [36]. Moreover, Nicolini-Panisson et al. conducted a cross-sectional study that included a total of 459 typically developing children and teenagers (227 boys and 232 girls) aged 3 − 18 years about normative values for the TUG test. In this study, they analyzed the reliability of the TUG test and found excellent intra-session and test–retest reliability. Similarly, to these findings, in the current study, we obtain a good test reliability an excellent inter-rater reliability in children with ASD.

According to the study by Nicolini-Panisson et al. [21], which displays the results of the amount of time spent performing the TUG test in young people with typical development stratified in the same age ranges as they are in the present study, we can see that the mean time is longer in young people with ASD. However, the sample used in the study by Nicolini-Panisson et al. [21] was taken from South Brazilian schoolchildren, and it was obtained using the TUG test with the modification that the children had to touch a target that was located 3 m away before they could sit back in the seat. Nonetheless, that variation was not used in the current investigation. As a result, we are unable to compare the results because the samples were drawn from different locations and used different protocols. However, Bustam et al. [40] examined the significance of the protocol choice and how it may affect the outcomes in 210 typically developing children between the ages of 6 and 12. We can observe that the average time required to complete the TUG test is shorter in typically developing children when we compare the data from that research using the same procedure employed in the current study in the same range of ages with our findings. Nevertheless, these results can only be compared for ages 6 to 12 and not for ages 13 to 18. Future research should compare the time spent on the TUG test in typically developing children with children with ASD in in all age ranges and using the same protocol.

When comparing two separate measurement systems, observers, or sessions to determine the degree of agreement in a data set, Bland–Altman plots are an effective tool [32, 33]. When we analyze the findings of the current study using Bland–Altman graphs, we noticed that neither the replicates nor the examiners' results showed any indication of bias. Additionally, the LOAs between examiners and test replicates were close, indicating that there was little variation between measurements. In line with these findings, Nicolini-Panisson et al. [21] also examined the Bland–Altman plots and found that 96% of the changes were contained inside the LOA, proving the TUG test's great reproducibility.

Williams et al. [25] found that the three original units of measurement generated for the three trials at time 1 had a SEM of 0.6 s and 0.4 s, respectively, and that the three trials at time 2 showed a SEM of 0.4 s. Furthermore, the magnitude of the SEM was typically less than 10%, expressed as a percentage of the mean scores. Considering that all SEM values for each age group were less than 10%, these SEM findings are consistent with those of the study by Williams et al. [25].

The current study has some limitations that should be highlighted to be fixed in futures lines of investigation. Firstly, it is important to emphasize that participants were chosen using a non-probabilistic sampling method from a series of people who met the inclusion requirements up until the calculated sample size was reached. A professional chose the participants based on predetermined criteria, such as availability and study interest, and non-random techniques. A non-probabilistic sampling technique could result in sample bias and restrict the findings’ generalizability to a larger base population. Secondly, the present study does not examine whether children who display greater impairment within the autism spectrum disorder have greater deficits in balance and gait because data on the degree of ASD impairment were not obtained. To determine if there is a relationship or not, future research should collect data on the level of ASD affectation in the different areas.

What is more, no relationship with other rating scales was developed because the primary goal of the study was to examine the reliability of the TUG test in children and adolescents with ASD. The present study does not identify the crucial areas that could indicate the risk of falls and worse dynamic balance, even while it is true that high values of the TUG test would suggest worse functional mobility and low values would indicate better functional mobility. To establish cut-off points indicating potential deficits in dynamic balance and gait and hence provide external validity to the test, future research should concentrate on comparing the test findings with other validated assessment scales.

However, based on our findings, it is reasonable to draw the conclusion that the TUG test can be used in children and teenagers with ASD because of its strong intra- and inter-rater reliability values, low proportion of measurement errors, and lack of significant bias based on by test repetition.

## Abbreviations

ASD: Autism spectrum disorder
CP: Cerebral palsy
DSM-5: Diagnostic and Statistical Manual of Mental Disorders
ICC: Interclass correlation coefficient
SD: Diff inter-rater differences
LOA: Limits of agreement
MDC: Minimum detectable change
TUG: Timed up and go test
SD: Standard deviation
SEM: Standard error of the measurement
